# Predicting postpartum diastasis recti abdominis using early-pregnancy abdominal wall ultrasound radiomics

**DOI:** 10.3389/fmed.2026.1879674

**Published:** 2026-09-29

**Authors:** Bei Li, Zhengfang Xu, Xiaoxue Zhao, Hao Sun, Aiping Zhu, Lu Sun

**Affiliations:** 1 Department of Ultrasound, Zhenjiang Ruikang Hospital, Zhenjiang, Jiangsu, China; 2 Department of Obstetrics, Affiliated Hospital of Jiangsu University, Zhenjiang, Jiangsu, China; 3 Department of Ultrasound, Affiliated Hospital of Jiangsu University, Zhenjiang, Jiangsu, China; 4 Department of Preventive Medicine, School of Medicine, Jiangsu University, Zhenjiang, Jiangsu, China; 5 Department of Ultrasound, Taizhou Hospital of Traditional Chinese Medicine, Taizhou, Jiangsu, China

**Keywords:** diastasis recti abdominis, early pregnancy, linea alba, radiomics, rectus abdominis, ultrasound

## Abstract

**Background:**

Diastasis recti abdominis (DRA) affects abdominal wall stability and is associated with low back pain and pelvic floor dysfunction. Early identification of high-risk individuals is important for prevention and individualized management.

**Objective:**

To develop and validate a clinical–radiomics model based on early-pregnancy two-dimensional grayscale ultrasound images for predicting DRA at 42 days postpartum and establishing a risk stratification system.

**Methods:**

This prospective study included 428 pregnant women at 5–8 gestational weeks, randomly divided into training (*n* = 300) and testing (*n* = 128) cohorts. Radiomic features were extracted from ultrasound images of the rectus abdominis and linea alba to construct Radscore1 and Radscore2. These scores were integrated with early-pregnancy BMI and exercise habits to develop a combined model. Model performance was evaluated using AUC, 10-fold cross-validation, Hosmer–Lemeshow test, calibration curves, and decision curve analysis. A risk scoring system was constructed from regression coefficients. Spearman correlation analysis assessed associations with postpartum inter-rectus distance (IRD).

**Results:**

Radscore1, Radscore2, early-pregnancy BMI, and regular exercise were independently associated with DRA, with regular exercise showing a protective association. The combined model achieved AUCs of 0.892 and 0.859 in the training and testing cohorts, respectively. In the scoring system, BMI ≥24 kg/m², absence of exercise habits, Radscore1 ≥ 2.73, and Radscore2 ≥ 1.85 were assigned 1, 1, 3, and 1 points, respectively. DRA incidence increased significantly with risk level (trend test, *P* < 0.001). Radscore1, Radscore2, early-pregnancy BMI, and rectus abdominis thickness were positively correlated with postpartum IRD.

**Conclusion:**

The clinical–radiomics model and risk scoring system accurately predicted DRA at 42 days postpartum and enabled early risk stratification, providing a quantitative basis for individualized intervention and follow-up. This is a single-center study; the findings require validation in independent multicenter cohorts before clinical implementation.

## 1 Introduction

Diastasis recti abdominis (DRA) is a common pregnancy- and postpartum-related abdominal wall dysfunction characterized by widening of the inter-rectus distance and stretching of the linea alba (1, 2). Beyond its cosmetic implications, DRA is characterized by widening of the inter-rectus distance and alterations in abdominal wall morphology, and may be associated with functional symptoms in some women (3, 4). Previous studies have suggested that core muscle training before or during early pregnancy may help reduce the risk of postpartum DRA (5, 6). However, as gestation progresses, the abdominal wall gradually approaches its maximal physiological expansion, thereby narrowing the window for effective preventive intervention. Therefore, early identification of women at high risk of developing DRA is clinically important for timely prevention, individualized counseling, and targeted postpartum follow-up.

The development of DRA is multifactorial. In addition to demographic and clinical factors such as age, body mass index (BMI), and exercise habits, the structural properties and biomechanical adaptability of the rectus abdominis and linea alba may play a crucial role in determining abdominal wall resilience during pregnancy (7, 8). Current risk assessment mainly relies on clinical variables and conventional ultrasound parameters, including BMI, rectus abdominis thickness, and inter-rectus distance (IRD). Although these indicators reflect the overall mechanical load or macroscopic morphological changes of the abdominal wall, they may be insufficiently sensitive to detect subtle tissue heterogeneity and functional reserve during early pregnancy (9). Two-dimensional ultrasound and shear wave elastography (SWE) have provided additional approaches for abdominal wall evaluation (10). Nevertheless, in the early gestational stage, conventional morphological parameters often show limited discriminatory ability, while SWE remains affected by region-of-interest selection, operator dependence, and insufficient parameter standardization, which may restrict its application in early risk prediction.

Radiomics enables the extraction of high-dimensional quantitative features from routine medical images, including texture, intensity, and morphological characteristics, thereby providing a non-invasive method to capture tissue microstructural heterogeneity (11). Compared with conventional measurements such as muscle thickness or IRD, radiomic analysis based on two-dimensional grayscale ultrasound images may offer a more comprehensive assessment of latent structural alterations in the rectus abdominis and linea alba (12). Importantly, these two anatomical structures have distinct but complementary biomechanical roles in the development of DRA. The rectus abdominis contributes to trunk stability and force transmission, whereas the linea alba maintains midline continuity and distributes mechanical load across the abdominal wall (13). Therefore, combined radiomic assessment of the rectus abdominis and linea alba may be more physiologically and biomechanically meaningful than evaluation of either structure alone, and may improve the accuracy of early-pregnancy risk stratification.

Accordingly, this study aimed to extract radiomic features from two-dimensional grayscale ultrasound images of the rectus abdominis and linea alba obtained during early pregnancy, construct rectus abdominis- and linea alba-derived radiomic scores, and integrate these scores with key clinical variables to develop a clinical–radiomics combined model for predicting DRA at 42 days postpartum. Furthermore, we sought to establish an interpretable risk scoring system for early identification of high-risk individuals, providing a quantitative basis for individualized intervention and follow-up management during pregnancy and the postpartum period.

## 2 Materials and methods

### 2.1 Study population

This was a single-center prospective cohort study. Pregnant women who consecutively attended Jiangsu University Affiliated Hospital between January 2022 and December 2024 and underwent abdominal wall ultrasound examination at 5–8 gestational weeks were enrolled. Baseline clinical data and ultrasound images were obtained during early pregnancy, and outcome assessment was performed at 42 days postpartum. The primary outcome of this study was diastasis recti abdominis (DRA) at 42 days postpartum. All participants underwent assessment of inter-rectus distance (IRD) at the umbilical level at 42 days postpartum, and DRA was defined as an IRD >2.0 cm. The inclusion criteria were as follows: primiparous women with singleton pregnancy; completion of two-dimensional grayscale abdominal wall ultrasound examination at 5–8 gestational weeks; completion of postpartum ultrasound follow-up at 42 days; and availability of complete clinical and imaging data. The exclusion criteria were as follows: previous abdominal surgery; congenital abdominal wall deformity; trunk abnormalities that might affect abdominal wall structure or function, such as scoliosis or neuromuscular disease; poor image quality unsuitable for radiomics analysis; or missing key clinical or imaging data. A total of 450 pregnant women were initially enrolled. After applying the inclusion and exclusion criteria, 428 participants were finally included in the analysis and randomly divided into a training cohort (*n* = 300) and a testing cohort (*n* = 128) at a ratio of 7:3 (Figure 1). The study was approved by the Ethics Committee of the hospital (the approval number SWYXLL20200429-8). Written informed consent was obtained from all participants.

**Figure 1:**
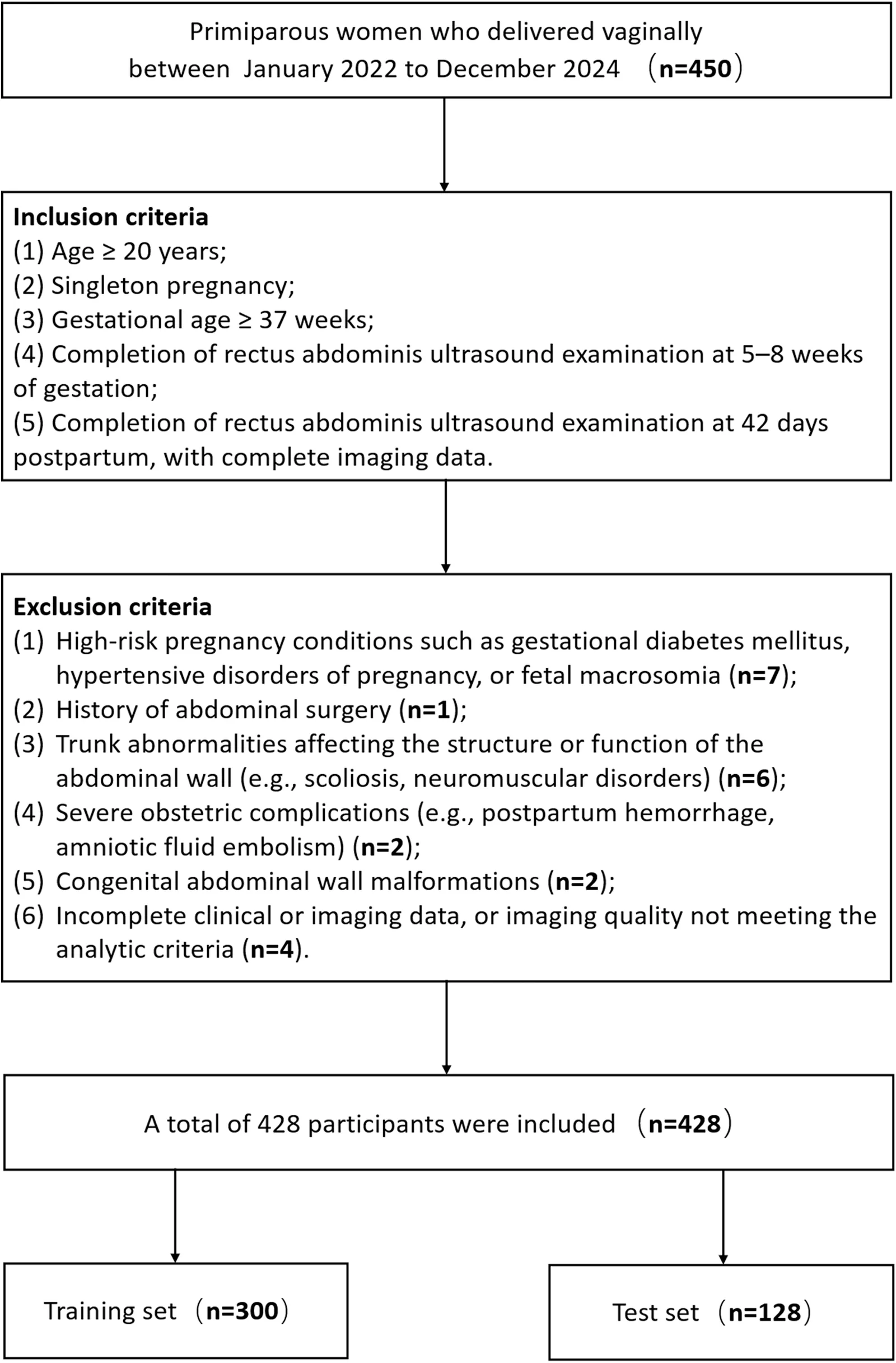
Study flowchart of participant selection and model development.

### 2.2 Demographic and laboratory data

Demographic and clinical data were collected, including age, height, body weight, and body mass index (BMI) at 5–8 gestational weeks. Early-pregnancy BMI was calculated based on height and body weight measured during the same period. Exercise habits were recorded at enrollment based on participant self-report. Regular exercise was defined as structured physical activity performed before pregnancy or during early pregnancy at least 2–3 times per week, with each session lasting no less than 30 min. Eligible activities included brisk walking, jogging, swimming, cycling, yoga, Pilates, or other structured exercise programs. Participants were therefore classified as having or not having regular exercise habits. Laboratory indicators included fasting blood glucose, glycated hemoglobin, total cholesterol, and triglycerides, all of which were measured before 12 gestational weeks.

### 2.3 Ultrasound examination and measurements

All ultrasound examinations were performed using a Mindray Resona 8S ultrasound system equipped with an L14-5WU linear-array transducer, with a frequency range of 4.0–14.0 MHz. Images were acquired under the superficial or musculoskeletal preset, with the scanning depth set at 4 cm and the two-dimensional grayscale gain maintained between 40 and 60 dB. All participants underwent baseline abdominal wall ultrasound examination at 5–8 gestational weeks and follow-up ultrasound examination at the same anatomical level at 42 days postpartum. Two-dimensional grayscale images were uniformly acquired in the transverse plane at the umbilical level. Images of the rectus abdominis and linea alba were obtained separately. For the rectus abdominis, the thickness and width of the left and right muscles were measured three times, and the average values were used for analysis. For the linea alba, thickness was measured in the same plane and repeated three times, with the average value recorded. At 42 days postpartum, the inter-rectus distance (IRD) was measured at the same umbilical level and used for outcome determination (Figure 2A). All images were stored in DICOM format for subsequent radiomics analysis. All ultrasound images used for radiomics analysis were selected according to predefined image quality criteria, including clear visualization of anatomical boundaries, complete display of the target region, and absence of substantial artifacts. Image selection was performed before outcome assessment, and sonographers were blinded to postpartum DRA status. Detailed information regarding participant preparation, image acquisition protocol, image selection criteria, and quality control procedures is provided in Supplementary Material S1.

**Figure 2:**
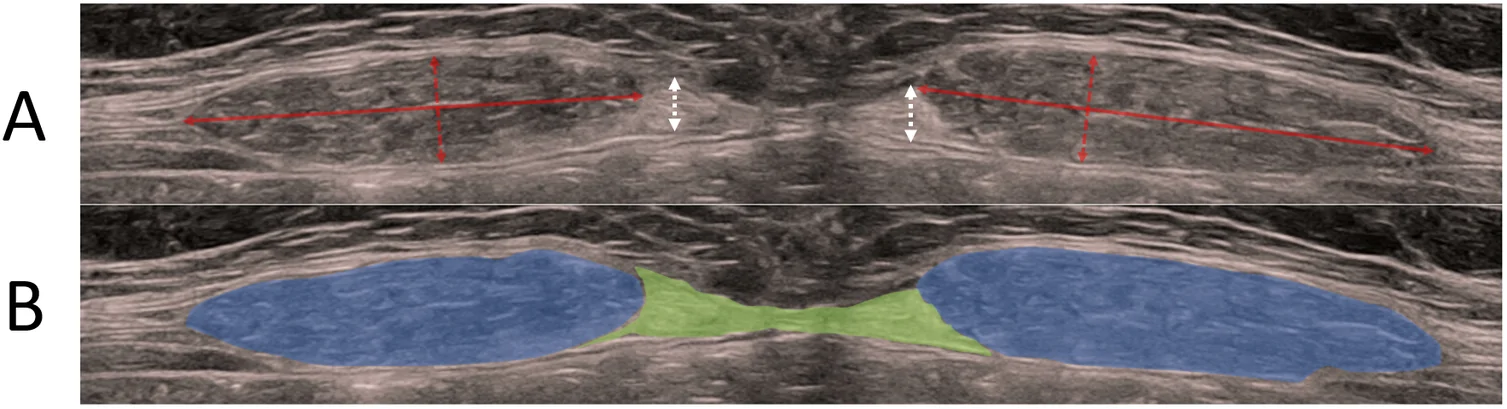
Ultrasound assessment and radiomics ROI delineation of the rectus abdominis and linea alba. **(A)** Panoramic two-dimensional ultrasound image showing the measurement of abdominal wall parameters. Rectus abdominis width was measured along the longitudinal axis, and rectus abdominis thickness was measured at the thickest point along the perpendicular short axis, with the mean value of both sides used for analysis. Linea alba thickness was measured at the both sides along the perpendicular short axis, and the mean of the three measurements was recorded. **(B)** Representative delineation of radiomics regions of interest. The blue region indicates the ROI of the rectus abdominis, and the green region indicates the ROI of the linea alba.

### 2.4 ROI delineation and radiomics feature extraction

Two-dimensional grayscale ultrasound images acquired during early pregnancy were imported into ImageJ for ROI delineation. Two experienced sonographers independently delineated the ROIs while blinded to postpartum outcomes. The rectus abdominis ROI was manually delineated along the visible muscle boundaries, whereas the linea alba ROI was delineated within the hyperechoic midline region at the umbilical level. ROI segmentation reproducibility was assessed using the Dice similarity coefficient, and features with poor reproducibility were excluded before model development. Radiomic features were extracted using PyRadiomics version 3.0 based on the original grayscale images and corresponding ROIs. Before feature extraction, images underwent intensity normalization and gray-level discretization; image resampling was disabled to preserve original resolution and avoid introducing spurious texture information through interpolation. To ensure reliability in the small linea alba ROI, we configured mask validation with a minimum ROI size of 50 pixels, enabled the skip-nans option to automatically exclude features returning NaN, and reviewed VoxelNum to exclude features from ROIs with insufficient voxel counts. Extracted features included first-order statistical features and texture features derived from gray-level co-occurrence matrix, gray-level run-length matrix, gray-level size zone matrix, neighboring gray-tone difference matrix, and gray-level dependence matrix. To reduce feature instability and redundancy, reproducible features were first retained according to intraclass correlation coefficients. Highly correlated features were subsequently removed using Spearman correlation analysis. Least absolute shrinkage and selection operator regression combined with the Boruta algorithm was then applied for further feature selection in the training cohort. Given the relatively small size of the linea alba ROI, only features demonstrating acceptable reproducibility were retained for subsequent analysis to minimize potential instability caused by limited ROI dimensions. Finally, radiomic scores for the rectus abdominis and linea alba were constructed separately and defined as Radscore1 and Radscore2. Detailed ROI definition, preprocessing parameters, feature extraction settings, reproducibility assessment, and feature selection procedures are provided in Supplementary Material S2.

### 2.5 Development of the risk scoring system

A simplified weighted risk-scoring system was developed from the predictors retained in the final multivariable logistic regression model. Continuous predictors were categorized according to the cutoff values determined in the preceding analyses. The absolute value of the smallest regression coefficient among the predictors included in the scoring model was used as the reference corresponding to 1 point. Relative weights were calculated by dividing the absolute regression coefficient of each predictor by the reference coefficient and rounding the resulting value to the nearest integer. For predictors with a negative regression coefficient, the complementary category representing the absence of the corresponding protective factor was used for score construction, and the absolute value of the original regression coefficient was used to calculate its relative weight.

### 2.6 Statistical analysis

Statistical analyses were performed using R software, version 4.3, and SPSS software, version 26.0. The normality of continuous variables was assessed using the Shapiro–Wilk test. Normally distributed continuous variables are presented as mean ± standard deviation, whereas non-normally distributed variables are presented as median with interquartile range. Categorical variables are presented as numbers and percentages. For between-group comparisons, independent-samples t tests, one-way analysis of variance, or Mann–Whitney U tests were used for continuous variables, as appropriate. Categorical variables were compared using the chi-square test. Clinical variables that differed significantly between groups, together with radiomic scores, were included in multivariable logistic regression analysis to construct the clinical–radiomics combined prediction model. Model discrimination was assessed using the area under the receiver operating characteristic curve. Model stability, goodness of fit, calibration performance, and clinical net benefit were evaluated using 10-fold cross-validation, the Hosmer–Lemeshow test, calibration curves, and decision curve analysis, respectively. The optimal cutoff values for continuous variables were determined using the Youden index derived from receiver operating characteristic curve analysis. The incidence of DRA across different risk levels was compared using the chi-square test, and trend analysis was performed using the Cochran–Armitage trend test for DRA incidence. Spearman correlation analysis was used to assess the associations between selected indicators and postpartum IRD. The Bonferroni method was applied for multiple-comparison correction. All statistical tests were two-sided, and *P* < 0.05 was considered statistically significant.

## 3 Results

### 3.1 Comparison of baseline characteristics

In the training cohort, 188 participants were classified into the DRA group and 112 into the non-DRA group. Significant differences were observed between the two groups in age, early-pregnancy BMI, and exercise habits (*P* < 0.05). In contrast, early-pregnancy rectus abdominis thickness, fasting blood glucose, glycated hemoglobin, total cholesterol, and triglyceride levels did not differ significantly between groups (*P* > 0.05). Detailed baseline characteristics are presented in Table 1.

**Table 1:** Comparison of baseline characteristics of participants in the training set.

Group		DRA (*n* = 188)	Non-DRA (*n* = 112)	t/*χ*2	*P* Value
Age (years, $\bar{x}\pm s$)		31.79 ± 4.50	30.69 ± 3.56	2.236	0.026
Early-pregnancy BMI (kg/m², $\bar{x}\pm s$)		22.16 ± 3.03	21.44 ± 2.56	2.155	0.035
Early-pregnancy rectus abdominis thickness (mm, $\bar{x}\pm s$)		10.17 ± 1.07	10.22 ± 0.97	−0.371	0.711
Early-pregnancy rectus abdominis width (mm, $\bar{x}\pm s$)		43.72 ± 6.38	43.91 ± 6.41	−0.223	0.824
Early-pregnancy linea alba thickness (mm, $\bar{x}\pm s$)		2.04 ± 0.48	2.01 ± 0.46	0.506	0.614
Fasting glucose (mmol/L, $\bar{x}\pm s$)		4.72 ± 0.55	4.69 ± 0.51	0.447	0.655
Glycated hemoglobin (%,$\bar{x}\pm s$)		5.23 ± 0.37	5.21 ± 0.35	0.434	0.665
Total cholesterol (mmol/L, $\bar{x}\pm s$)		4.62 ± 0.81	4.59 ± 0.77	0.297	0.767
Triglycerides (mmol/L, $\bar{x}\pm s$)		1.35 ± 0.48	1.33 ± 0.45	0.335	0.738
Regular exercise [n (%)]	Yes	28 (14.9%)	30 (26.8%)	4.312	0.038
	No	160 (85.1%)	82 (73.2%)		

DRA, diastasis recti abdominis; BMI, body mass index.

### 3.2 Reproducibility analysis and feature selection

The Dice similarity coefficient for repeated rectus abdominis ROI delineation by the same operator was 0.90 (95% CI: 0.87–0.93). The Dice similarity coefficients for inter-operator delineation of the rectus abdominis ROI and linea alba ROI were 0.89 (95% CI: 0.86–0.92) and 0.86 (95% CI: 0.82–0.90), respectively. Radiomic features extracted from the left and right rectus abdominis ROIs showed high consistency, with an ICC of 0.93 (95% CI: 0.89–0.97).

A total of 1,820 and 1,524 radiomic features were extracted from the rectus abdominis and linea alba ROIs, respectively. After reproducibility-based filtering, correlation-based redundancy reduction, and feature selection using LASSO regression combined with the Boruta algorithm, six key rectus abdominis features and five key linea alba features were retained. Based on these features, the rectus abdominis radiomics score and linea alba radiomics score were constructed and defined as Radscore1 and Radscore2, respectively (Figure 3). In the training cohort, both Radscore1 and Radscore2 were higher in the DRA group than in the non-DRA group. Between-group comparisons showed significant differences for Radscore1 (t = −3.32, *P* = 0.001) and Radscore2 (t = −2.42, *P* = 0.016).

**Figure 3:**
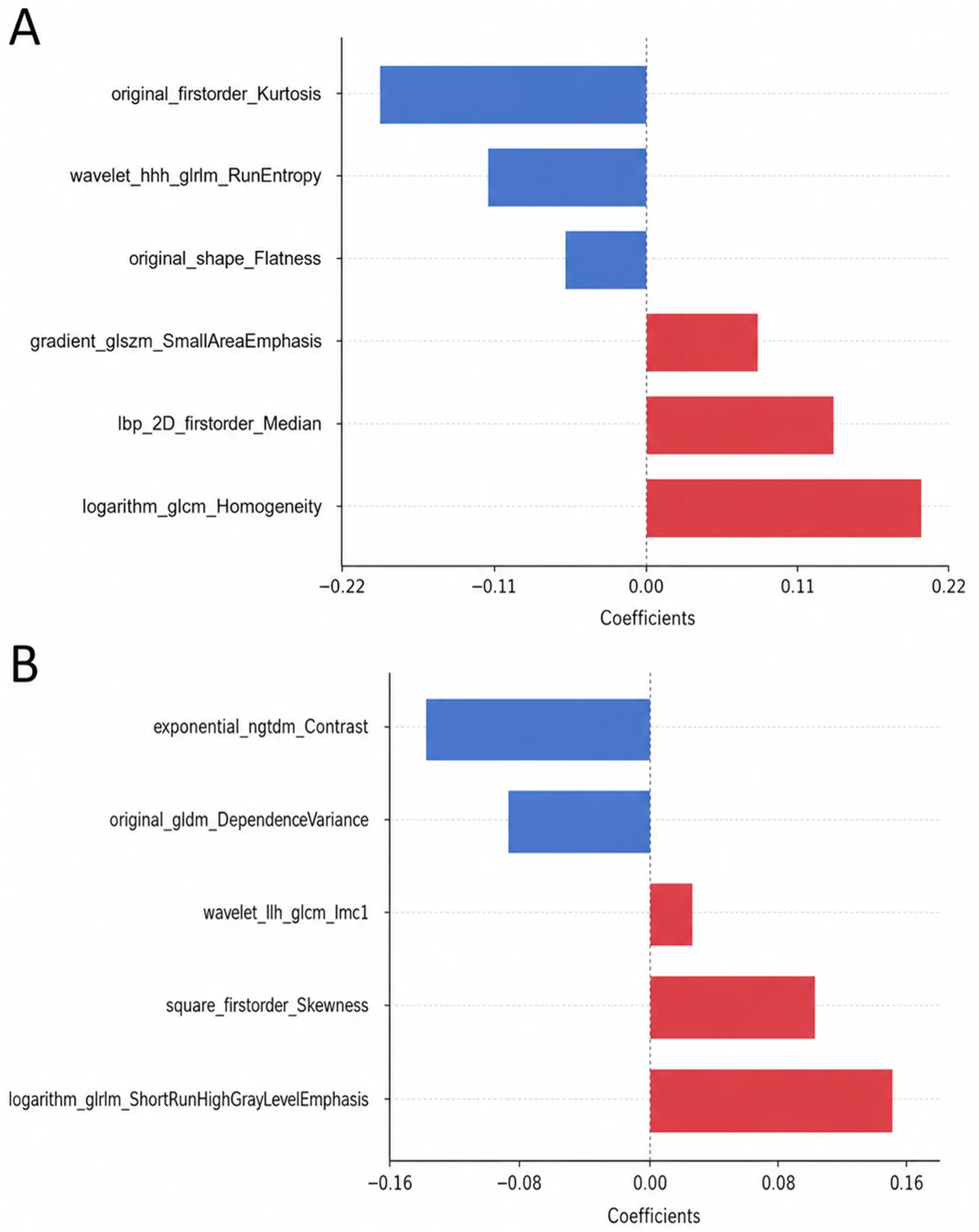
Feature weight maps of rectus abdominis and linea alba radiomics. **(A)** Distribution of feature weights for Radscore1 (rectus abdominis). **(B)** Distribution of feature weights for Radscore2 (linea alba).

### 3.3 Construction and validation of the combined model

We constructed a clinical model incorporating age, early-pregnancy BMI, and exercise habits; a radiomics model incorporating Radscore1 and Radscore2; and a combined model incorporating both clinical and radiomics variables (Table 2). In the training cohort, the AUCs of the clinical, radiomics, and combined models were 0.784, 0.862, and 0.892, respectively; in the testing cohort, the corresponding AUCs were 0.761, 0.832, and 0.859. DeLong's test demonstrated that the combined model significantly outperformed the clinical model in both cohorts (training: *P* < 0.001; testing: *P* = 0.005) and the radiomics model (training: *P* = 0.021; testing: *P* = 0.033). In the testing cohort, the addition of the radiomics score to the clinical variables yielded an NRI of 0.40 (*P* = 0.001) and an IDI of 0.12 (*P* < 0.001). Further internal validation of the combined model showed that ten-fold cross-validation yielded a mean AUC of 0.875 (95% CI: 0.853–0.897). The Hosmer–Lemeshow test indicated good model fit (training cohort: *χ*² = 6.956, *P* = 0.542; testing cohort: *χ*² = 6.528, *P* = 0.581). The calibration curves demonstrated good agreement between the predicted probabilities and the observed outcomes. Decision curve analysis further confirmed that the model provided a positive net clinical benefit across a relatively wide range of threshold probabilities (Figure 4).

**Table 2:** Multivariable regression analysis of the clinical–radiomics combined model.

Variable	*β* Coefficient	OR	95% CI	*P* Value
Clinical model				
Age	0.094	1.101	1.042–1.174	0.002
Early-pregnancy BMI	0.281	1.325	1.173–1.490	<0.001
Exercise habit	−0.462	0.630	0.442–0.832	0.011
Radiomics model				
Radscore1	1.062	2.892	1.983–3.806	<0.001
Radscore2	0.812	2.252	1.554–3.062	<0.001
Combined model				
Radscore1	0.512	1.669	1.312–1.933	<0.001
Radscore2	0.209	1.233	1.026–1.507	0.003
Early-pregnancy BMI	0.188	1.207	1.004–1.411	0.011
Regular exercise	−0.185	0.831	0.705–0.994	0.021

Radscore1, Radiomics score of the rectus abdominis; Radscore2, Radiomics score of the linea alba; BMI, body mass index.

**Figure 4:**
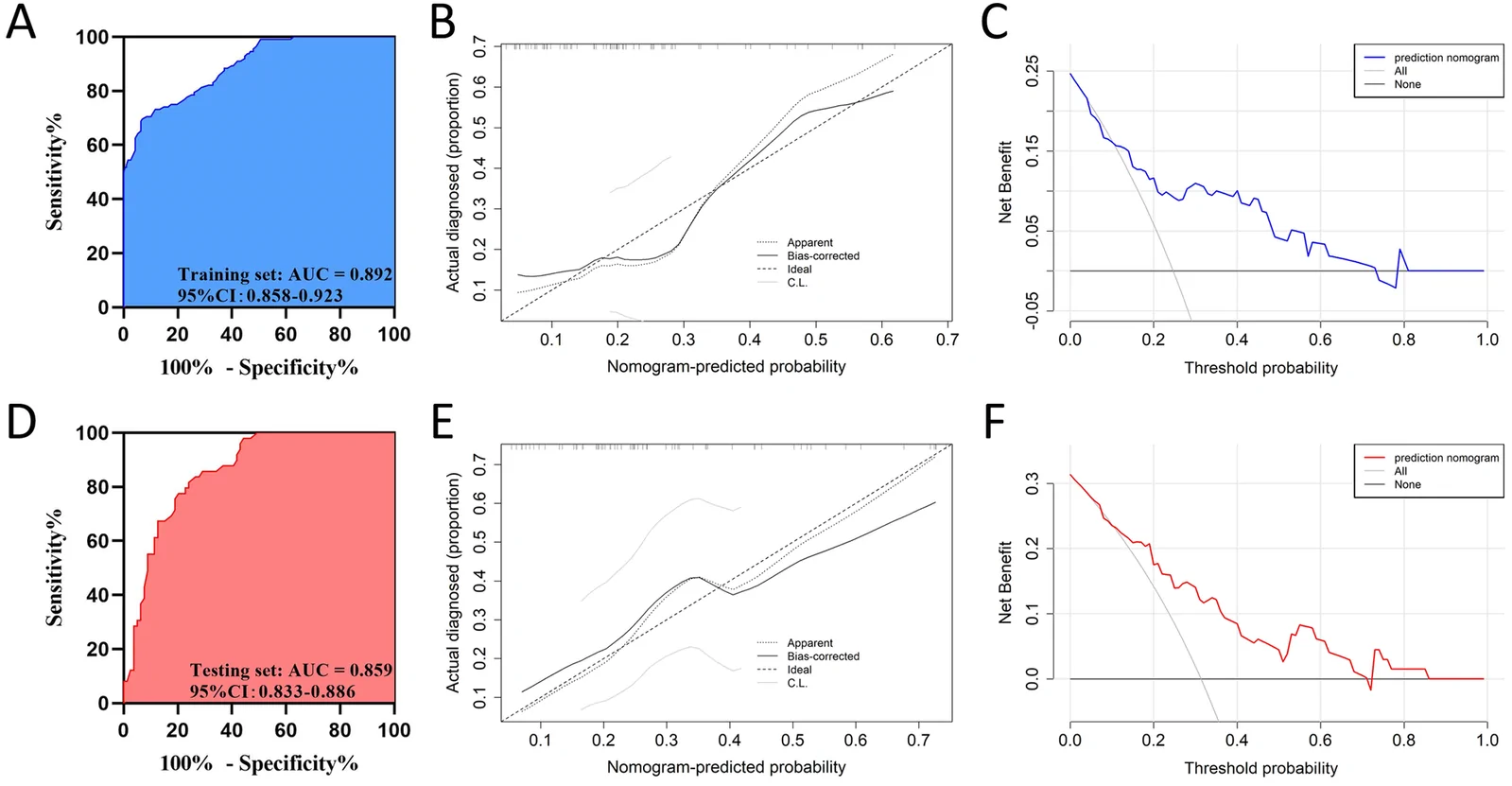
Performance evaluation of the radiomics model in the training and test sets. **(A–C)** Model performance in the training set: **(A)** receiver operating characteristic curve, **(B)** calibration curve comparing predicted probabilities of DRA with observed frequencies, and **(C)** decision curve analysis evaluating clinical utility across different threshold probabilities. **(D–F)** Model performance in the test set: **(D)** receiver operating characteristic curve, **(E)** calibration curve, and **(F)** decision curve analysis. ROC, receiver operating characteristic.

### 3.4 DRA risk scoring system and risk stratification

Based on the Youden index of the ROC curves, the optimal cutoff values for Radscore1 and Radscore2 were 2.73 and 1.85, respectively. The resulting point assignments were 1 point for early-pregnancy BMI ≥24 kg/m², 1 point for absence of regular exercise, 3 points for Radscore1 ≥ 2.73, and 1 point for Radscore2 ≥ 1.85. The scoring criteria are shown in Table 3, and the detailed derivation of the point assignments is provided in Supplementary Table S1. Participants were stratified into three risk categories according to the total score: 0–2, 3–4 and 5–6 points. In the training cohort, the incidence rates of DRA across these categories were 18.8% (6/32), 38.0% (38/100) and 85.7% (74/168), respectively. In the test cohort, the corresponding incidence rates were 15.0% (3/20), 39.6% (19/48) and 88.3% (53/60), respectively. Chi-square tests showed significant differences in DRA incidence among the risk categories in both the training and test cohorts (both *P* < 0.001). Trend tests further demonstrated a progressive increase in DRA incidence with increasing risk score in both cohorts (both *P* < 0.001).

**Table 3:** Weighted scoring system for DRA risk prediction.

Variable	Scoring criterion	Weighted score
Early-pregnancy BMI (≥24 kg/m² = 1, <24 kg/m² = 0)	≥24 kg/m²	1
Absence of regular exercise (absent = 1, present = 0)	Absent	1
Radscore1 (≥2.73 = 1, <2.73 = 0)	≥2.73	3
Radscore2 (≥1.85 = 1, <1.85 = 0)	≥1.85	1

BMI, body mass index.

### 3.5 Correlation analysis of IRD and its stratified distribution

Spearman correlation analysis showed that Radscore1, Radscore2, early-pregnancy BMI, and early-pregnancy rectus abdominis thickness were positively correlated with IRD at 42 days postpartum (all *P* < 0.05), with correlation coefficients of 0.72, 0.63, 0.60, and 0.66, respectively. After Bonferroni correction for multiple comparisons involving four tests, these associations remained statistically significant at the corrected threshold of *P* < 0.0125 (Figure 5A). Based on the risk categories derived from the scoring system, IRD at 42 days postpartum increased progressively with higher risk categories in both the training and test cohorts. The differences among risk categories were statistically significant in both cohorts (training cohort: F = 5.067, *P* < 0.001; test cohort: F = 4.198, *P* < 0.001), with significant linear trends observed in both datasets (trend test, *P* < 0.001) (Figure 5B).

**Figure 5:**
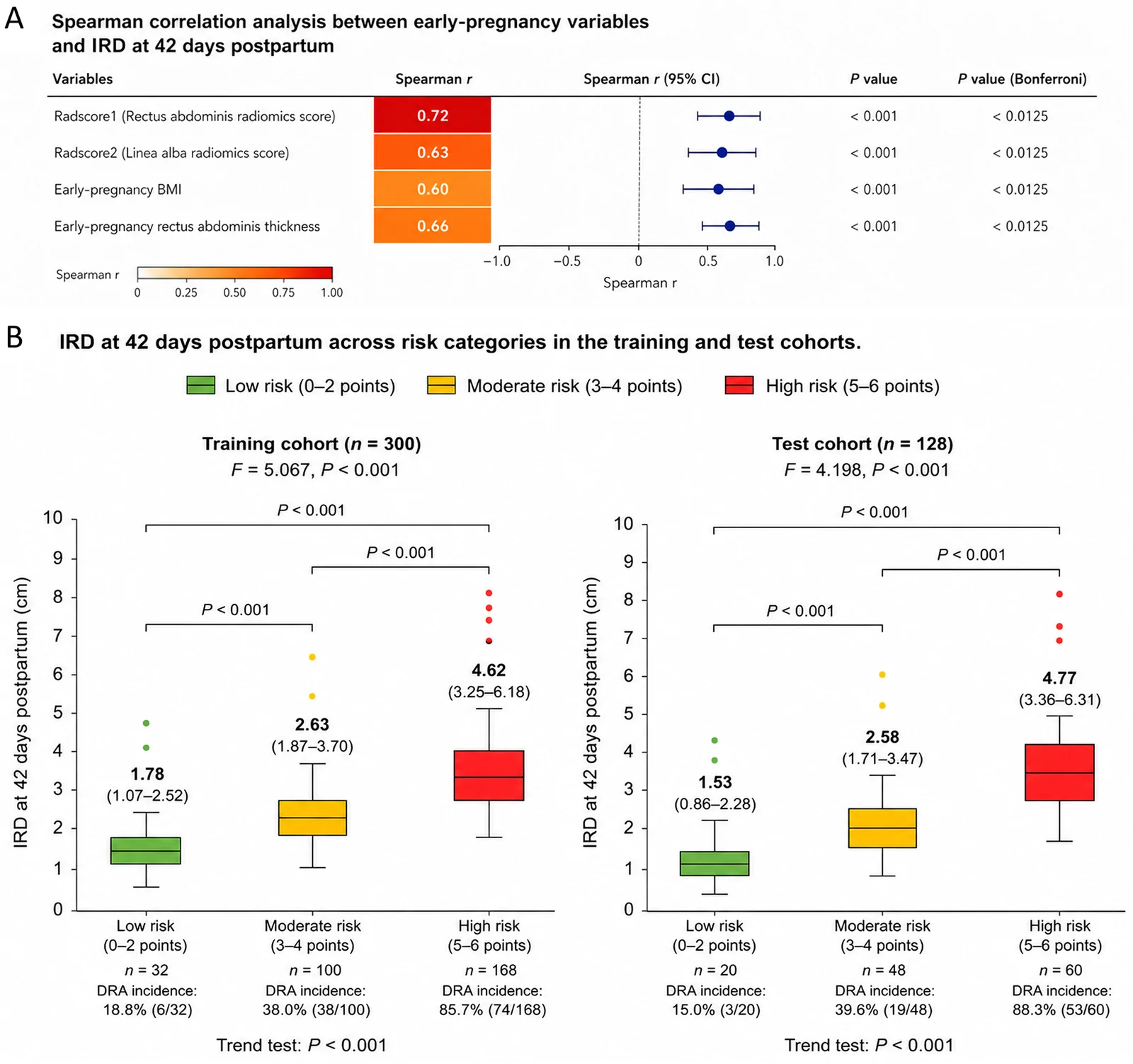
Correlation analysis and stratified distribution of postpartum inter-rectus distance (IRD). **(A)** Spearman correlation analysis between postpartum IRD and Radscore1, Radscore2, early-pregnancy BMI, and early-pregnancy rectus abdominis thickness. **(B)** Distribution of postpartum IRD across risk categories derived from the scoring system in the training and test sets. IRD, inter-rectus distance; BMI, body mass index.

## 4 Discussion

In this study, radiomic features of the rectus abdominis and linea alba were jointly extracted from two-dimensional grayscale ultrasound images acquired during early pregnancy. Based on these features, we developed a combined clinical-radiomics model to predict the risk of DRA at 42 days postpartum and further established a risk scoring system. The results showed that Radscore1, Radscore2, early-pregnancy BMI, and exercise habits were independent factors associated with DRA. The combined model demonstrated good discriminative ability and calibration performance in both the training and test cohorts. Moreover, the risk scoring system enabled effective stratification of pregnant women, supporting the feasibility of early DRA risk assessment based on ultrasound-derived imaging information obtained during early pregnancy.

From a biomechanical perspective, the development of DRA is not only related to increased intra-abdominal pressure during pregnancy but also involves alterations in abdominal wall adaptation. The rectus abdominis plays an important role in trunk stability and force transmission, whereas the linea alba maintains midline continuity and load distribution (14). Previous studies have suggested that DRA is not merely characterized by widening of the inter-rectus distance but also reflects structural alterations of abdominal wall morphology that may be associated with functional symptoms (8, 15). Lax et.al proposed that the occurrence of DRA may be associated with an imbalance in transverse abdominal wall tension (16). Consistent with this hypothesis, our radiomics analysis suggests that image-derived tissue heterogeneity in the rectus abdominis and linea alba may already be detectable during early pregnancy and is closely associated with the subsequent risk of DRA at 42 days postpartum.

The findings of this study differ from those obtained using conventional clinical assessment methods. Early-pregnancy rectus abdominis thickness and metabolic indicators, such as fasting blood glucose and glycated hemoglobin, did not differ significantly between the DRA and non-DRA groups, suggesting that these routine clinical variables may have limited predictive value for DRA risk (9, 17). In contrast, radiomic features can capture subtle tissue-level differences, particularly structural heterogeneity of the rectus abdominis and linea alba, thereby providing a potential imaging biomarker for early identification of DRA risk (7, 18). Unlike the study by Miasek et al, which evaluated rectus abdominis thickness and IRD using ultrasound, the present study incorporated comprehensive radiomic information from both the rectus abdominis and linea alba (19). This approach may provide a more complete assessment of abdominal wall adaptation and yield more informative indicators than single-dimensional thickness measurements alone.

Successful implementation of radiomics depends on the accuracy and reproducibility of image segmentation (20, 21). In this study, we evaluated the reproducibility of ROI delineation and the consistency of radiomic features extracted from the bilateral rectus abdominis muscles (22). The Dice similarity coefficients for both rectus abdominis and linea alba ROIs were favorable, and the ICC for radiomic features extracted from the left and right rectus abdominis ROIs was high. These findings support the feasibility of this method under a standardized workflow. Nevertheless, previous studies have indicated that the reproducibility and external validation of radiomics remain challenging. Yamashita et al. noted that although manual segmentation can achieve acceptable repeatability in small-sample analyses, operator-dependent variability may still affect results in large-scale clinical datasets (23). Therefore, automated or semi-automated segmentation methods should be considered in future studies to further improve segmentation efficiency and consistency.

The combined model demonstrated that radiomic features provided additional information beyond conventional clinical variables (8, 20). In multivariable analysis, Radscore1 and Radscore2 remained independently associated with DRA after adjustment for early-pregnancy BMI and exercise habits, suggesting that image-derived tissue heterogeneity of the abdominal wall has independent predictive value for DRA. This finding is consistent with the study by Abdullah et.al, which suggested that microscopic or tissue-level alterations in the rectus abdominis may precede clinically visible structural changes (24). Therefore, early monitoring of these changes may be important for DRA prevention. However, the external validity and long-term predictive performance of the proposed model still require confirmation in larger cohorts.

Unlike most previous studies that focused on postpartum assessment or the effects of a single intervention, the present study shifted the timing of risk assessment to early pregnancy and integrated radiomic features from both the rectus abdominis and linea alba (25). Although postpartum IRD is a key diagnostic indicator of DRA, risk identification after delivery has limited preventive value. Previous studies have shown that postpartum abdominal training may reduce IRD, but its effect on persistent DRA remains inconsistent (26). By using ultrasound-based radiomics during early pregnancy, this study not only identified early image-derived tissue alterations but also provided a potential basis for early intervention and individualized follow-up during pregnancy. However, it should be noted that IRD may continue to decrease during subsequent postpartum months. Therefore, the present model predicts early postpartum DRA at 42 days rather than persistent DRA. Future longitudinal studies with longer follow-up periods are needed to determine whether early pregnancy radiomic features can predict long-term persistence of DRA.

This study has practical clinical implications. The proposed risk scoring system may provide a simple tool for early risk stratification and may support targeted monitoring and counseling. By identifying high-risk individuals during early pregnancy, this scoring system could support more targeted clinical monitoring and optimize preventive care. However, it should be emphasized that although the model performed well in the present cohort, its generalizability remains to be validated because the data were derived from a single center and have not yet been tested across different populations, ultrasound systems, or acquisition settings. External validation should therefore be a priority in future research.

Several limitations should be acknowledged. First, this was a single-center study with a relatively limited sample size, and external validation is urgently needed. Second, ROI delineation relied on manual segmentation. Although segmentation reproducibility was assessed, potential operator-related bias cannot be fully excluded. Future studies may incorporate automated or semi-automated segmentation techniques. In addition, although acquisition protocols were standardized, the stability of radiomic features under variations in ultrasound acquisition conditions was not systematically evaluated, which should be further investigated in future studies. Third, only ultrasound images acquired at the umbilical level were analyzed; future studies should consider whether imaging findings differ across other anatomical levels of the abdominal wall. Fourth, exercise habits were obtained through self-report and were categorized as a binary variable. Therefore, recall bias and misclassification cannot be completely excluded. Future studies incorporating quantitative assessment of exercise intensity, duration, and adherence are warranted. Fifth, although this study focused on ultrasound imaging data obtained during early pregnancy, the development of DRA is multifactorial. Although the model showed satisfactory performance in internal validation, external geographic validation using independent populations and different ultrasound systems is required before clinical application.

In conclusion, a combined clinical-radiomics model based on early-pregnancy ultrasound radiomic features of the rectus abdominis and linea alba, together with early-pregnancy BMI and exercise habits, demonstrated good discrimination and calibration for predicting the risk of DRA at 42 days postpartum. The derived risk scoring system provided a quantitative basis for early risk stratification, with its generalizability pending further external validation.

## Data Availability

The raw data supporting the conclusions of this article will be made available by the authors, without undue reservation.
