# Supplementary material for: Predicting postpartum diastasis recti abdominis using early-pregnancy abdominal wall ultrasound radiomics
Source: Front Med (Lausanne). 2026 Sep 29;13:1879674. doi: 10.3389/fmed.2026.1879674 (PMC13657946; doi:10.3389/fmed.2026.1879674)
Supplement: Supplementary file 1 [file Table1.docx]

**Supplementary Material S1**

**Detailed Ultrasound Acquisition, Image Selection, and Quality Control**

All ultrasound examinations were performed by two uniformly trained sonographers. During the examination, participants were placed in a relaxed supine position, with both arms crossed over the chest and both lower limbs naturally extended. The abdominal region from the xiphoid process to the pubic symphysis was fully exposed. During image acquisition, participants were instructed to maintain natural breathing and avoid breath-holding, the Valsalva maneuver, or any other actions that could markedly alter intra-abdominal pressure. The transducer was placed perpendicular to the abdominal wall surface, and only minimal necessary pressure was applied to reduce compression-related deformation of tissue morphology and anatomical boundaries. Baseline examinations were performed at 5–8 gestational weeks, and follow-up examinations at 42 days postpartum were conducted using the same body position, the same transducer, and a consistent acquisition protocol to improve measurement reproducibility. The transverse plane at the umbilical level was selected as the standardized imaging plane. This plane is a common site for DRA assessment and reflects abdominal wall structural changes under intra-abdominal pressure. The rectus abdominis and linea alba were evaluated in the same imaging plane.

For the rectus abdominis, images were required to include the target muscle and its anterior and posterior sheath boundaries as completely as possible, with panoramic imaging used when necessary to ensure full visualization. The thickness and width of the left and right rectus abdominis muscles were each measured three times and averaged. For the linea alba, thickness was measured in the same plane while avoiding obvious periumbilical acoustic shadowing and posterior attenuation whenever possible; measurements were obtained at the thickest portions on both sides, repeated three times for each side, and averaged. At the 42-day postpartum follow-up, IRD was measured at the same umbilical level. If multiple valid measurements were obtained for the same participant, the average value was used as the final measurement.

Images included in the subsequent radiomics analysis were required to meet the following quality control criteria: clear visualization of target structure boundaries; complete display of the key anatomical region; absence of obvious motion artifacts; and absence of severe acoustic shadowing or posterior attenuation obscuring the target region. If multiple frames were acquired from the same participant and anatomical site, the frame with the clearest boundary, fewest artifacts, and most complete structural display was selected for ROI delineation and feature extraction. Image selection was performed before reviewing postpartum IRD measurements, and the operators responsible for image selection were blinded to postpartum DRA outcomes. Images with incomplete boundaries or artifacts that substantially affected interpretation were excluded from the radiomics analysis. All eligible images were stored in DICOM format and managed using a standardized naming and storage protocol for subsequent radiomics processing.

**Supplementary Material S2**

**ROI Delineation, Preprocessing, and Radiomics Workflow**

All early-pregnancy two-dimensional grayscale ultrasound images included in the radiomics analysis were imported into ImageJ software. Two sonographers with experience in abdominal wall ultrasound independently delineated the ROIs while blinded to postpartum outcomes. The rectus abdominis ROI was delineated on transverse ultrasound images at the umbilical level. The ROI was drawn to cover the identifiable rectus abdominis muscle tissue as completely as possible, while avoiding obvious subcutaneous tissue, regions outside the rectus sheath, and areas with significant artifacts. The linea alba ROI was delineated in the hyperechoic midline region at the umbilical level. Because of the relatively small thickness of the linea alba, only regions with relatively clear boundaries, continuous echogenicity, and minimal interference from periumbilical acoustic shadowing or posterior attenuation were included. Features affected by insufficient ROI size or poor reproducibility were excluded during the feature filtering process. Images with incomplete boundaries, severe artifacts obscuring the target structure, or unstable anatomical boundary identification were excluded from ROI analysis. To assess the reproducibility of ROI segmentation, the Dice similarity coefficient was calculated for repeated delineations by the same operator and for delineations between different operators. To further evaluate the feasibility of using unilateral rectus abdominis features for radiomics analysis, 30 participants were randomly selected from the training cohort. ROIs were delineated separately on the left and right rectus abdominis, radiomic features were independently extracted, and feature consistency between both sides was subsequently assessed.

Radiomic feature extraction was performed using PyRadiomics version 3.0. The same image processing and feature extraction workflow was applied to both the training and testing cohorts. Feature extraction was based on the original two-dimensional grayscale images and corresponding ROIs. Extracted features included first-order statistical features and multiple texture feature classes, including gray-level co-occurrence matrix, gray-level run-length matrix, gray-level size zone matrix, neighborhood gray-tone difference matrix, and gray-level dependence matrix features. These features were used to quantify the microstructural imaging characteristics of the rectus abdominis and linea alba from multiple dimensions, including grayscale distribution, texture heterogeneity, and spatial organization.

To reduce the impact of measurement variability and redundant features on model stability, intraclass correlation coefficients were first calculated for features extracted by different operators. Features with an intraclass correlation coefficient greater than 0.85 were considered reproducible and were retained for subsequent analysis. Spearman correlation analysis was then used to remove highly correlated redundant features, with a threshold of |r| > 0.75, to reduce information duplication and multicollinearity.

Subsequently, least absolute shrinkage and selection operator regression was performed in the training cohort to further reduce the feature space, and the Boruta algorithm was applied to identify features most strongly associated with the outcome. Finally, based on the selected features and their corresponding regression coefficients, radiomic scores were constructed separately for the rectus abdominis and linea alba, defined as Radscore1 and Radscore2, respectively. These scores were then incorporated into subsequent model development.

**Technical handling of small ROIs in PyRadiomics**

Given the small thickness of the linea alba ROI (approximately 2 mm), we implemented several technical measures within PyRadiomics (version 3.0) to ensure feature extraction reliability:

Mask validation: The minimum-ROI-Dimensions parameter was set to 2 (the default for 2D ROIs), and minimum-ROI-Size was set to 50 pixels. ROIs with dimensions or pixel counts below these thresholds were excluded from feature extraction.

Resampling: Image resampling was disabled (resampled-Pixel-Spacing: None). This decision was made because resampling (particularly upsampling) could artificially interpolate pixel values and introduce spurious texture information that does not exist in the original image, potentially compromising feature validity in small ROIs.

Handling of NaN values: PyRadiomics may return NaN for certain texture features when the ROI contains too few voxels to construct a valid texture matrix (e.g., insufficient voxel pairs for GLCM or GLRLM computation). We enabled the skip-nans option during batch extraction, which automatically removed features returning NaN from the output.

Voxel count monitoring: The VoxelNum parameter—indicating the number of voxels in the ROI after preprocessing—was reviewed for each extracted feature. Features originating from ROIs with insufficient voxel counts were excluded before subsequent reproducibility analysis and model construction.

These technical measures, combined with the ICC-based reproducibility filtering (ICC > 0.85) described above, ensured that only stable and reliably computed features were retained for Radscore2 construction.

**Supplementary Table S1**

**Supplementary Table S1** Derivation of the weighted DRA risk score

| Predictor | Coding for score construction | Regression coefficient (β) | Relative weight (β/0.185) | Assigned points |
| --- | --- | --- | --- | --- |
| Early-pregnancy BMI ≥24 kg/m² | 1 = yes,  0 = no | 0.188 | 1.016 | 1 |
| Absence of regular exercise | 1 = absent,  0 = present | 0.185* | 1.000 | 1 |
| Radscore1≥2.73 | 1 = yes, 0 = no | 0.512 | 2.768 | 3 |
| Radscore2≥1.85 | 1 = yes, 0 = no | 0.209 | 1.130 | 1 |

Note: In the corrected combined model, regular exercise was coded as 1 = presence and 0 = absence, yielding a regression coefficient of −0.185. For score construction, this protective factor was expressed as the complementary risk factor, absence of regular exercise, and its coefficient was therefore represented by its absolute value (0.185). The smallest absolute regression coefficient (0.185) was used as the reference corresponding to 1 point. Relative weights were calculated by dividing each absolute coefficient by 0.185 and rounding to the nearest integer. BMI: body mass index
